# Scrub typhus mimicking Parkinson’s disease

**DOI:** 10.1186/s13104-015-1428-x

**Published:** 2015-09-15

**Authors:** Ranjan Premaratna, S. H. Nuwan Chamara Wijayalath, J. K. N. Dhanushka Miththinda, N. K. B. K. R. G. Wijesinghe Bandara, H. Janaka de Silva

**Affiliations:** Colombo North Teaching Hospital, Ragama, Sri Lanka

**Keywords:** Scrub typhus, Parkinson, *Orientia tsutsugamushi*

## Abstract

**Background:**

Scrub typhus is a re-emerging infection in Sri Lanka. It often poses a diagnostic challenge and tends to present as a febrile illness of uncertain origin. Undiagnosed illness may progress to serious multi-systemic complications. Here we report a case of scrub typhus presenting with features of Parkinsonism.

**Case presentation:**

A 62-year-old previously healthy Sri Lankan native male from the Western province of Sri Lanka presented with high fever with malaise, myalgia and arthralgia for 17 days. On the 5th day of illness he developed intermittent resting tremor in his right arm and leg associated with stiffness, difficulty in carrying out normal work and difficulty in smiling. He denied similar previous episodes. There were no other associated neurological manifestations. Clinical examination revealed a high amplitude low frequency resting tremor in his right hand, a mask-like face and increased muscle tone limited to the right side with normal reflexes. The rest of the system examination was normal except for an eschar over the abdomen. His investigations revealed lymphocytic leukocytosis, high erythrocyte sedimentation rate and immunofluorescence assay-IgM and IgG against *Orientia tsutsugamushi* Karp antigen were positive with rising titers. With oral doxycycline and azithromycin his fever settled within 48 h and a complete recovery of Parkinson’s features was observed within 2 weeks.

**Conclusion:**

Doctors practicing in endemic regions should be familiar with delayed clinical manifestations of scrub typhus and should carefully look for an eschar in order to avoid delay in the diagnosis.

**Electronic supplementary material:**

The online version of this article (doi:10.1186/s13104-015-1428-x) contains supplementary material, which is available to authorized users.

## Background

Rickettsiae comprise a diverse collection of gram negative pleomorphic intracellular organisms with several antigenic differences. While mammals such as rodents and arthropods are natural hosts of rickettsioses, they are usually transmitted to humans by arthropods [1, 2]. Therefore the epidemiology of human diseases caused by rickettsiae is intimately related to the presence of the vector that transmits it. The clinical severity and duration of illnesses associated with different rickettsial infections vary considerably, even within a given antigenic group. Rickettsioses range in severity from diseases that are usually relatively mild (rickettsial pox, cat scratch disease, and African tick-bite fever) to those that can be life-threatening (epidemic typhus, Rocky Mountain spotted fever, and Oroya fever), and they vary in duration from those that can be self-limiting to chronic or recrudescent (Brill–Zinsser disease) [1–3]. Most patients with rickettsial infections recover with timely use of appropriate antibiotic therapy such as doxycycline, chloramphenicol, azithromicin and rifampicin.

Rickettsial illnesses, caused by organisms within the genus of rickettsiae, are divided into three biogroups; spotted fever group, typhus group and scrub typhus group. The organisms within the scrub typhus group differ strikingly from Rickettsial species of the spotted fever and typhus groups. The three major serotypes of *Orientia tsutsugamushi*are Karp, Gilliam, and Kato.

**Fig. 1:**
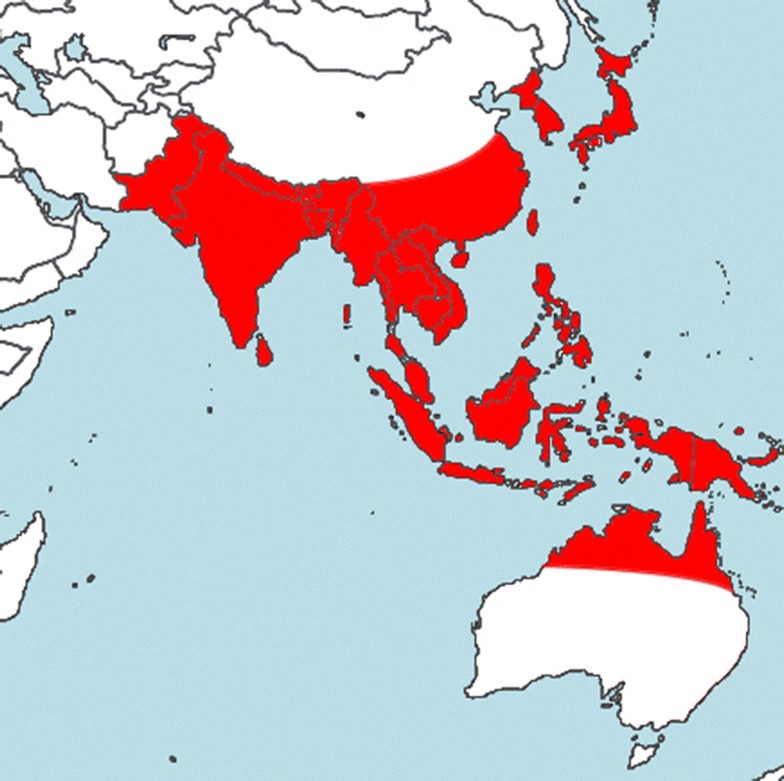
Scrub typhus endemic regions of the world (map drawan by the authors based on published data on scrub typhus epidemiology) [16]; Commonly endemic areas are highlighted in* red*

Scrub typhus caused by *O. tsutsugamushi* is a seasonal vector borne infectious disease. It is prevalent in the tsutsugamushi triangle of South East Asia (Fig. 1) and the western Pacific region [4]. It is considered a re-emerging infection in Sri Lanka [5, 6]. Scrub typhus presents with an acute febrile illness with varying other non-specific symptoms and signs such as headache, malaise, body aches, cough and lymphadenopathy. Presence of an eschar helps in the clinical diagnosis [4]. However, it often poses a diagnostic challenge and tends to present as a febrile illness of uncertain origin [7]. Although the illness is known to recover without treatment in a considerable number of patients, delay in the diagnosis is known to cause multi-system involvement and serious complications such as encephalitis, myocarditis, acute renal failure and multi-organ failure [4, 5, 8]. Here we report a case of scrub typhus presenting with features of Parkinsonism.

## Case presentation

**Fig. 2:**
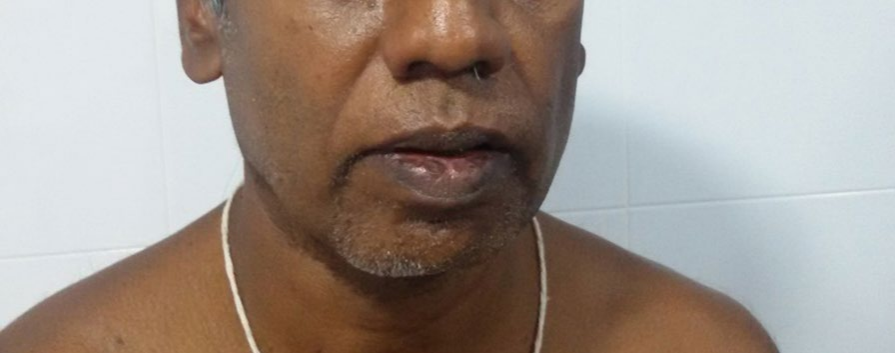
Facial appearence at presentation

**Fig. 3:**
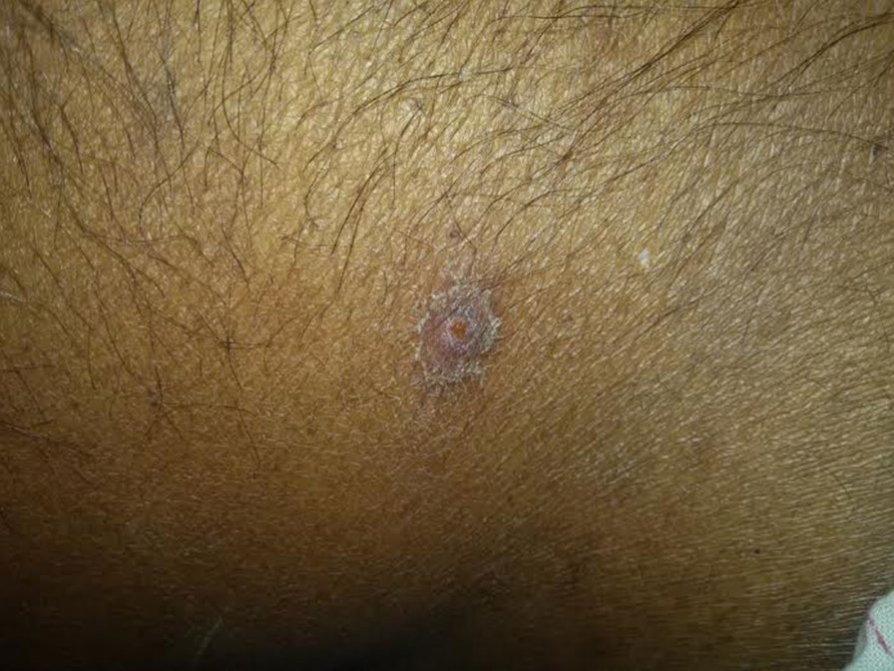
Eschar

A 62-year-old previously healthy Sri Lankan native male from Gampaha, in the Western Province of Sri Lanka presented with high fever associated with chills and rigors for 17 days. He also complained of malaise, myalgia and arthralgia for the same duration but denied any urinary, respiratory or abdominal symptoms. Until he presented to us, he had obtained treatment froma local hospital for the illness with no clinical improvement. He had received co-amoxyclav, clarythromicin and paracetamol for 4 days. He had not been consuming any medication prior to the illness and had not received any drug that would result in extrapyramidal features. Around the 5th day of the clinical illness he had developed intermittent resting tremor in his right arm and leg. By the time he presented to us he had stiffness and very frequent intermittent resting tremor (Additional file 1: Video). This resulted in difficulty to carry out normal work with the right hand. He also found it difficult to walk due to unusual stiffness and heaviness of the right leg. Furthermore, he found it difficult to smile with others and felt very distressed. He denied similar previous episodes. There was no associated hearing impairment, seizures, or altered level of consciousness. There were no symptoms to suggest involvement of cerebellar system or autonomic nervous system. There was no family history of movement disorders. He had been working in his garden 7 days prior to the onset of fever and rest of the past medical history was unremarkable. Examination revealed intermittent high amplitude low frequency resting tremor in his right hand (Additional file 1: Video) and a mask like face where he found difficult to smile or show his teeth (Fig. 2). He also had increased muscle tone limited to the right side with normal tendon reflexes.The rest of the central nervous system was unremarkable. His blood pressure was 140/90 mmHg in both supine and standing positions, pulse rate 88 beats per minute and there were no cardiac murmurs. The respiratory system revealed few basal crackles and the abdominal examination was unremarkable except for a superficial crater like lesion (Fig. 3) suggestive of an eschar. He did not have lymphadenopathy or a rash.

**Fig. 4:**
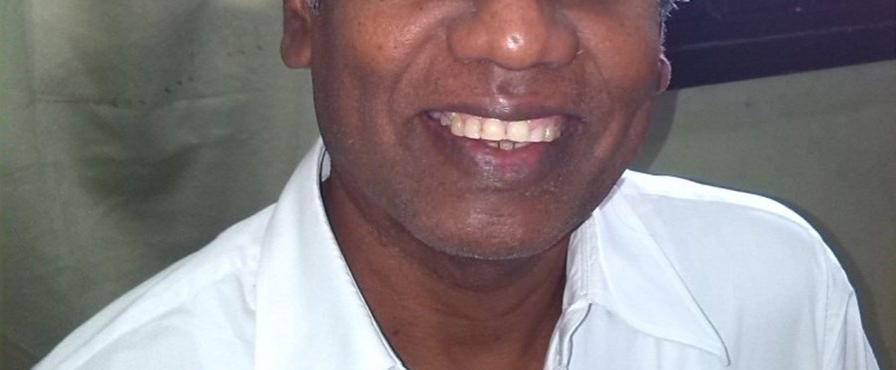
Facial appearence after recovery

His full blood count was 13.4 × 10^9^/L (Neutrophils 43 %, Lymphocytes 56 %), Erythrocyte sedimentation rate was 80 mm/1st h and had a normal urine analysis, liver and renal function tests. The CT scan of the brain and the Electroencephalogram were normal. We did not perform a lumbar puncture or an Magnetic resonance image scan of the brain. He was positive for Immunofluoresence Assay (IFA)-IgM and IgG for *O. tsutsugamushi* on the 17th day of illness and the IFA-IgG titre using Orientia Karp antigen was 1:1024 which rose up to 1:16,384 after 2 weeks confirming scrub typhus. His fever settled with oral doxycycline and azithromycin within 48 h and demonstrated some improvement in his Parkinsonism features prior to discharge from hospital in 4 days. He vistied for review 2 weeks after discharge and by this time he showed complete improvement and was happy with a smiling face (Fig. 4).

## Discussion

This previously healthy patientgradually developed unilateral rest tremor, rigidity and a masked face suggestive of Parkinson’s disease during an acute febrile illness. The development of symptoms was at a later stage of the illness and could well be due to delay in diagnosing scrub typhus. He had a dramatic improvement following treatment.

Various movement disorders such as Parkinsonism, may occur as a consequence of central nervous system infection, as either an acute or chronic manifestation [9, 10]. Similar involvement has been documented with scrub typhus [11–13]. These include Meningoencephalitis or encephalomyelitis with or without focal neurological signs, isolated cranial nerve palsies, opsoclonus, myoclonus, transient trigeminal neuralgia, brachial plexus neuropathy, post recovery Guillain–Barre syndrome, polyneuropathy along with cerebral infarction and acute disseminated encephalomyelitis [14]. Transient Parkinsonism with myoclonus in all four limbs has been previously described in one patient with scrub typhus [15]. Most descriptions of extra pyramidal rigidity associated with rickettsial infections highlight bilateral clinical manifestations [15]. Our patient had unilateral limb tremor and unilatelal rigidity mimicking Parkinson’s disease and had a dramatic recovery following treatment. He had an escar over the abdomen however it had been missed at an earlier stages of the illness.

## Conclusion

We suggest that doctors who practice in endemic regions should be familiar with delayed manifestations of scrub typhus and careful examination for eschar is the key to clinical diagnosis. Furthermore, a trial of anti-rickettsial treatment on empirical basis may prove beneficial for patients with “unusual and undiagnosed febrile syndromes” because response to treatment is dramatic in rickettsial infections. Such an approach would behelpful in settings whererickettsial disease diagnostic facilities are not available.

## Consent

Written informed consent was obtained from the patient for publication of this Case Report and any accompanying images. A copy of the written consent is available for review by the Editor-in-Chief of this journal.

## Additional file

**Additional file 1: Video.** High frequency intermittent tremo.

## Abbreviations

IFA: immunofluorescence assay
IgG: immunoglobulin type G
IgM: immunoglobulin type M
